# Effects of advanced platelet-rich fibrin combined with a collagen membrane on human periodontal ligament stem cells at two extract concentrations: an in vitro study

**DOI:** 10.1186/s12903-026-09064-1

**Published:** 2026-06-26

**Authors:** Meo Nguyen, Bao Quoc Minh Nguyen, Truong Thien Tran, Ha Le Bao Tran, Thu Ngoc Yen Nguyen, Thi Hoa Ho, Ha Viet Nguyen, Nam Cong-Nhat Huynh

**Affiliations:** 1 Faculty of Dentistry, University of Medicine and Pharmacy at Ho Chi Minh City, 652 Nguyen Trai, Cho Lon Ward, Ho Chi Minh City, 749000 Viet Nam; 2 Laboratory of Tissue Engineering and Biomedical Materials, Department of Physiology and Animal Biotechnology, Faculty of Biology-Biotechnology, University of Science, Vietnam National University, Ho Chi Minh City, Viet Nam

**Keywords:** Advanced platelet-rich fibrin, Collagen membrane, Human periodontal ligament stem cells, Cell viability, Metabolic activity, Wound closure

## Abstract

**Background:**

Advanced platelet-rich fibrin (A-PRF) is used in periodontal regeneration due to its autologous fibrin matrix and bioactive content. Collagen membranes are widely used in guided tissue regeneration. However, it remains unclear whether combining A-PRF with a collagen membrane alters early cellular responses. This in vitro study compared the effects of A-PRF alone and A-PRF combined with a collagen membrane on the viability, proliferation-related metabolic activity, and migration-related wound closure of human periodontal ligament stem cells (hPDLSCs) at two extract concentrations.

**Methods:**

hPDLSCs were exposed to extracts prepared from A-PRF alone or A-PRF combined with a porcine collagen membrane (A-PRF-Col) at 100% and 20% concentrations. Extracts were prepared using A-PRF derived from the blood of four donors. Cell viability was assessed after 24 h using an MTT assay and morphological observation. Proliferation-related metabolic activity was evaluated by MTT assay on days 1, 3, 5, and 7. Migration-related wound closure was evaluated using a scratch assay after 24 h. Data were analysed using repeated-measures ANOVA with post hoc multiple-comparison tests. *p* < 0.05 was considered statistically significant.

**Results:**

Early hPDLSC responses differed between A-PRF and A-PRF-Col and varied between the two extract concentrations. Based on mean relative growth rate values, only 100% A-PRF-Col met the predefined cytotoxicity criterion, whereas 100% A-PRF showed a borderline and donor-variable viability profile. Proliferation-related metabolic activity increased and peaked around day 5, but different concentrations and formulations affected the patterns. Migration-related wound closure was primarily influenced by extract concentration, with 20% extracts showing greater closure than their 100% counterparts. At 20% concentration, A-PRF achieved better closure than A-PRF-Col.

**Conclusions:**

Under the present cell-source and extract-preparation conditions, hPDLSC responses differed between A-PRF and A-PRF-Col and varied between the two tested extract concentrations. Diluted extracts generally elicited more favourable early cellular responses than undiluted extracts. The A-PRF-Col construct did not uniformly enhance A-PRF effects across endpoints. These findings should be interpreted as exploratory evidence that A-PRF-Col has an endpoint- and concentration-related biological profile that requires further optimisation.

**Trial registration:**

Not applicable.

**Supplementary Information:**

The online version contains supplementary material available at https://doi.org/10.1186/s12903-026-09064-1.

## Background

Periodontitis is a chronic inflammatory disease that damages tooth-supporting tissues, including the periodontal ligament and alveolar bone, and manifests as periodontal pockets, gingival recession, or both [1]. Although advances in periodontal treatment can help control inflammation and slow disease progression, predictable periodontal attachment apparatus regeneration still presents a challenge [2].

Guided tissue regeneration is a well-established periodontal regenerative strategy that uses a barrier membrane to prevent epithelial downgrowth and gingival connective tissue invasion while maintaining a space for tissue formation [3]. Among available barrier materials, collagen membranes are popular due to their biocompatibility, resorbability, and structural similarity to the extracellular matrix [4]. However, conventional collagen membranes mainly serve as barrier materials, and their capacity to actively provide biological stimulation sufficient to optimise early cellular regenerative responses may be limited [3, 5].

Platelet-rich fibrin (PRF) is a blood concentrate rich in fibrin, platelets, and leukocytes that aids in wound healing [6, 7]. Advanced platelet-rich fibrin (A-PRF), a PRF variant produced by low-speed centrifugation, can support growth factor release [6, 8]. These properties provide a rationale for combining A-PRF with collagen-based membranes as a biofunctionalisation strategy. Previous studies on PRF-treated collagen-based biomaterials have suggested that PRF can interact with collagen matrices and alter their biological activity, including growth factor presentation and angiogenesis-related responses [9–11]. However, the cellular effects of combining solid A-PRF with a collagen membrane on hPDLSCs remain insufficiently understood.

Human periodontal ligament stem cells (hPDLSCs) are valuable for periodontal tissue repair due to their clonogenicity, proliferative capacity, and differentiation potential [12]. They commonly show fibroblast-like morphology, express mesenchymal markers, and lack haematopoietic markers [13, 14]. These properties make hPDLSCs a relevant in vitro model for evaluating biomaterials and biological mediators intended for periodontal regeneration.

Previous in vitro studies have shown that PRF-based formulations can influence cellular processes relevant to wound healing and regeneration, including proliferation, migration, adhesion, and differentiation, although the reported effects vary depending on the PRF protocol, cell type, assay design, and exposure conditions [7, 15–17]. In our previous in vitro study, A-PRF combined with a xenogenic bone substitute material enhanced hPDLSC proliferation and migration, with effects varying across extract concentrations [18]. However, it remains unclear how A-PRF combined with a collagen membrane affects early hPDLSC responses and whether these effects differ between extract concentrations. Therefore, the aim of the present study was to compare the effects of A-PRF alone and A-PRF combined with a collagen membrane on hPDLSC viability, proliferation-related metabolic activity, and migration-related wound closure at 100% and 20% extract concentrations. We hypothesised that A-PRF combined with a collagen membrane would differ from A-PRF alone in its effects on early hPDLSC responses and that these effects would vary between the two tested extract concentrations.

## Methods

This donor-matched in vitro experimental study compared the effects of A-PRF alone and A-PRF combined with a collagen membrane on hPDLSC viability, proliferation-related metabolic activity, and migration-related wound closure at two extract concentrations: 100% and 20%.

### Human periodontal ligament stem cell culture

Human periodontal ligament stem cells (hPDLSCs) were obtained from the Laboratory of Tissue Engineering and Biomedical Materials, University of Science, Vietnam National University, Ho Chi Minh City. A single previously characterised hPDLSC donor-derived cell source was used. The cells were originally isolated by the outgrowth method, and the original tissue collection, isolation, and characterisation procedures were ethically approved and previously reported [19]. These cells had been previously characterised as periodontal ligament-derived cells with mesenchymal stromal cell-associated markers, including CD44, CD73, and CD90, with low expression of CD34, CD45, and HLA-DR, and were shown to undergo osteogenic and adipogenic differentiation in vitro [19]. hPDLSCs obtained at passage 3 were subcultured to passage 4, and passage 4 cells from the same cell source were used for all experiments. This approach was used to reduce cell-source variability across experimental conditions.

Cells were cultured in complete culture medium, consisting of Dulbecco’s Modified Eagle’s Medium/Nutrient Mixture F-12 (DMEM/F12; Sigma-Aldrich, St. Louis, MO, USA), supplemented with 10% fetal bovine serum (Sigma-Aldrich, St. Louis, MO, USA) and 1% penicillin-streptomycin solution (10,000 U/mL penicillin and 10 mg/mL streptomycin; Sigma-Aldrich, St. Louis, MO, USA), at 37 °C in a humidified atmosphere containing 5% CO₂. The medium was changed every 2 days.

### A-PRF extract and A-PRF with a collagen membrane extract preparation

Peripheral blood was collected from four healthy volunteers aged 20–30 years who were non-smokers, reported no regular alcohol consumption, had no systemic disease, were not taking any medications or dietary supplements, had no periodontal disease or active inflammatory condition, and had platelet counts within the institutional laboratory reference range. The study protocol was approved by the Biomedical Research Ethics Committee of the University of Medicine and Pharmacy at Ho Chi Minh City (Approval number 1973/ĐHYD-HĐĐĐ). All participants were informed about the study, and written informed consent was obtained prior to blood collection.

Blood was drawn into 10 mL glass A-PRF vacuum tubes without additives (16 × 107 mm; Process for PRF, Nice, France) and immediately centrifuged using the manufacturer’s pre-registered A-PRF preset on a PRF DUO Quattro centrifuge (Process for PRF, Nice, France). According to the manufacturer’s manual, the pre-registered A-PRF preset used in this study corresponds to 1300 rpm for 14 min. For the 10 mL tube configuration used, the reported rotor radius is 11 cm, giving a calculated RCF-max of approximately 208 × g. Previous PRF methodological standardisation work described the Duo centrifuge system as a fixed-angle centrifuge with approximately 40° rotor angulation, a clot-level radius of approximately 88 mm, and a maximum radius of approximately 110 mm [20]. After centrifugation, the A-PRF clot was separated from the red blood cell fraction and compressed for 30 s using a PRF Box^®^ (Process for PRF, Nice, France) to obtain an A-PRF membrane.

For the A-PRF combined with a collagen membrane group (A-PRF-Col), a sterile porcine collagen membrane (Geistlich Bio-Gide^®^, Geistlich Pharma AG, Wolhusen, Switzerland) with an original size of 30 × 40 mm was trimmed to 20 × 30 mm. The A-PRF clot was placed over the collagen membrane, and both materials were compressed together for 30 s using a PRF Box^®^ (Process for PRF, Nice, France), following a method adapted from Blatt et al. [10]. For all A-PRF-Col preparations, the collagen membrane size was kept constant, and the A-PRF clot mass was used to calculate the extraction ratio.

For extract preparation, each A-PRF membrane and A-PRF-Col construct was processed separately. The wet mass of each A-PRF membrane, or of the A-PRF component in each A-PRF-Col construct, was measured before incubation. The 100% extracts were prepared by incubating the materials in serum-free DMEM/F12 at an extraction ratio of 0.2 g/mL for 24 h at 37 °C under static conditions, following a protocol adapted from ISO 10993-12. In the A-PRF-Col group, the extraction ratio was calculated based on the wet mass of the A-PRF component to standardise the biological dose of A-PRF across formulations. After incubation, the conditioned media were centrifuged at 3,000 rpm (approximately 1,065 × g) for 5 min to remove residual cells and debris. The supernatants were collected and defined as 100% extracts. The 20% extracts were prepared separately under the same conditions, using an extraction ratio of 0.04 g/mL. These two extract conditions were selected based on previous PRF-derived exudate and conditioned-medium studies on periodontal ligament cells that tested 100% and 20% preparations, and were intended to compare higher and lower extract-load conditions rather than to establish a full dose-response relationship.

For each blood donor, paired A-PRF and A-PRF-Col preparations were generated, and extracts were prepared at both 100% and 20% concentrations. Thus, each donor contributed to all four experimental groups and was treated as one biological replicate (*n* = 4), allowing donor-matched comparisons across formulation and concentration.

### Effect of A-PRF and A-PRF-Col extracts on hPDLSC viability

The effects of A-PRF and A-PRF-Col extracts on hPDLSC viability were assessed using an MTT-based metabolic activity assay adapted from ISO 10993-5:2009. Basal serum-free DMEM/F12 was used as the negative control, and the basal serum-free DMEM/F12 with 20% DMSO was used as the positive control for assay validation and comparison.

hPDLSCs were seeded in 96-well plates at a density of 2 × 10^4 cells/well and cultured for 24 h at 37 °C in a humidified atmosphere containing 5% CO₂. The culture medium was then replaced with 100 µL of the corresponding extract or control medium. Four technical replicate wells were used for each group in each experiment. After 24 h of incubation, unstained microscopic images were captured using an inverted microscope (CKX53; Olympus, Tokyo, Japan) and CellSens imaging software (Olympus, Tokyo, Japan) to document overall cell density, attachment, distribution, and gross morphology.

The test media were removed and replaced with 100 µL of MTT solution (0.5 mg/mL; Sigma-Aldrich, St. Louis, MO, USA). Cells were incubated for 4 h under the same conditions. The MTT solution was then removed, and the resulting formazan crystals were dissolved in 100 µL of ethanol-DMSO solution (70:30, v/v). The plates were incubated for 30 min, and the contents of each well were mixed thoroughly by pipetting. Optical density was measured at 570 nm (OD_570_) in single-wavelength mode using an EZ Read 400 microplate reader (Biochrom Ltd., Cambridge, UK). No reference wavelength was applied. Condition-specific blank wells without cells were included for each extract and control condition and used for background correction.

Relative growth rate (RGR) was calculated as follows: RGR (%) = (mean blank-corrected OD_570_ of the experimental group / mean blank-corrected OD_570_ of the negative control group) × 100. According to ISO 10993-5:2009, samples with RGR values below 70% of the negative control were interpreted as meeting the predefined cytotoxicity criterion.

### Effect of A-PRF and A-PRF-Col extracts on hPDLSC proliferation-related metabolic activity

The effects of A-PRF and A-PRF-Col extracts on hPDLSC proliferation-related metabolic activity were assessed using an MTT assay at four time points. Basal serum-free DMEM/F12 was included as a serum-free baseline reference control and was used only for secondary control-based comparisons, not in the primary factorial analysis.

hPDLSCs were seeded in 96-well plates at a density of 2 × 10^4 cells/well and cultured for 24 h at 37 °C in a humidified atmosphere containing 5% CO₂ to allow cell attachment and spreading. The medium was then replaced with the corresponding extracts or control media. Four technical replicate wells were used for each group at each time point. Proliferation-related metabolic activity was assessed on days 1, 3, 5, and 7 using the MTT assay. Each time point used a separate 96-well plate with no medium replacement during the assay period. Condition-specific blank wells without cells were included for each extract and control condition for background correction. The MTT assay was performed as described in the viability assay section, and blank-corrected OD values at 570 nm were used for analysis.

### Effect of A-PRF and A-PRF-Col extracts on hPDLSC migration-related wound closure

Migration-related wound closure was evaluated using a scratch assay. hPDLSCs were seeded at a density of 3 × 10^5 cells/well in six-well plates and cultured until a near-confluent monolayer was achieved. The culture medium was then replaced with 3 mL of basal serum-free DMEM/F12, and the cells were incubated for 24 h at 37 °C in a humidified atmosphere containing 5% CO₂ to induce serum deprivation. After this period, the medium was removed, and a single linear scratch was created in each well using a sterile 100–1000 µL pipette tip. Detached cells were removed by washing once with 1× phosphate-buffered saline, and 3 mL of the corresponding extract or control medium was immediately added to each well. Basal serum-free DMEM/F12 and complete culture medium were included as serum-free baseline and growth-supporting reference controls, respectively. These reference controls were not included in the primary factorial analysis. Four technical replicate wells were used for each donor-derived extract and condition.

Images of the scratched area were captured at baseline (0 h) and after 24 h using an Olympus CKX-RCD inverted microscope equipped with a DP2-BSW microscopy camera at 4× objective magnification. The underside of each plate was marked to define the same field imaged at both time points, and one field per well was analysed. Wound closure was quantified using ImageJ 1.52a software by a blinded examiner and expressed as the percentage of wound closure relative to baseline, calculated as [(A0 − A24) / A0] × 100, where A0 and A24 represent the cell-free area at 0 and 24 h, respectively. Technical replicate values were averaged to obtain one donor-level value for statistical analysis.

Because scratch closure reflects a composite response influenced by collective migration, cell spreading, and proliferation-related activity, this outcome was interpreted as migration-related wound closure rather than direct measurement of cell migration alone.

### Statistical analysis

Statistical analyses were performed using GraphPad Prism 10 (GraphPad Software, San Diego, CA, USA). The blood donor-derived extract was treated as the matched biological unit. Four technical replicate wells were averaged before analysis, yielding one donor-level value per condition. Data are presented as mean ± standard deviation of donor-level values unless otherwise stated.

Model assumptions were evaluated descriptively using residual plots and Q–Q plots. For within-donor factors with more than two levels, sphericity was not assumed, and Greenhouse–Geisser-corrected degrees of freedom and p-values were reported. No a priori sample size calculation was performed because this was an exploratory in vitro study using a fixed number of donor-derived biological replicates.

For the viability assay, blank-corrected OD_570_ values from the four experimental extract groups were analysed using two-way repeated-measures ANOVA, with formulation and concentration as within-donor factors, followed by Šídák-adjusted pairwise comparisons. RGR values were used for descriptive cytotoxicity assessment.

For proliferation-related metabolic activity, blank-corrected OD_570_ values from the four experimental extract groups were analysed using three-way repeated-measures ANOVA, with time, concentration, and formulation as within-donor factors. Prespecified simple-effects analyses were then performed using two-way repeated-measures ANOVA within each concentration and within each formulation, followed by Šídák-adjusted pairwise comparisons at each time point. As a secondary control-based analysis, the four extract groups and the serum-free baseline reference control were analysed across time using two-way repeated-measures ANOVA, followed by Dunnett-adjusted comparisons of each extract group versus the serum-free baseline reference control at each time point.

For migration-related wound closure, wound closure percentages across the four experimental extract groups were analysed using a two-way repeated-measures ANOVA with formulation and concentration as within-donor factors, followed by Šídák-adjusted pairwise comparisons. As a secondary control-based analysis, wound closure percentages were compared with the serum-free baseline reference control using repeated-measures one-way ANOVA followed by Dunnett’s multiple-comparison test. Assay-specific reference controls were included for validation and interpretation, but were not included in the primary factorial models. *p* < 0.05 was considered statistically significant.

## Results

### Effect of A-PRF and A-PRF-Col extracts on hPDLSC viability

**Fig. 1:**
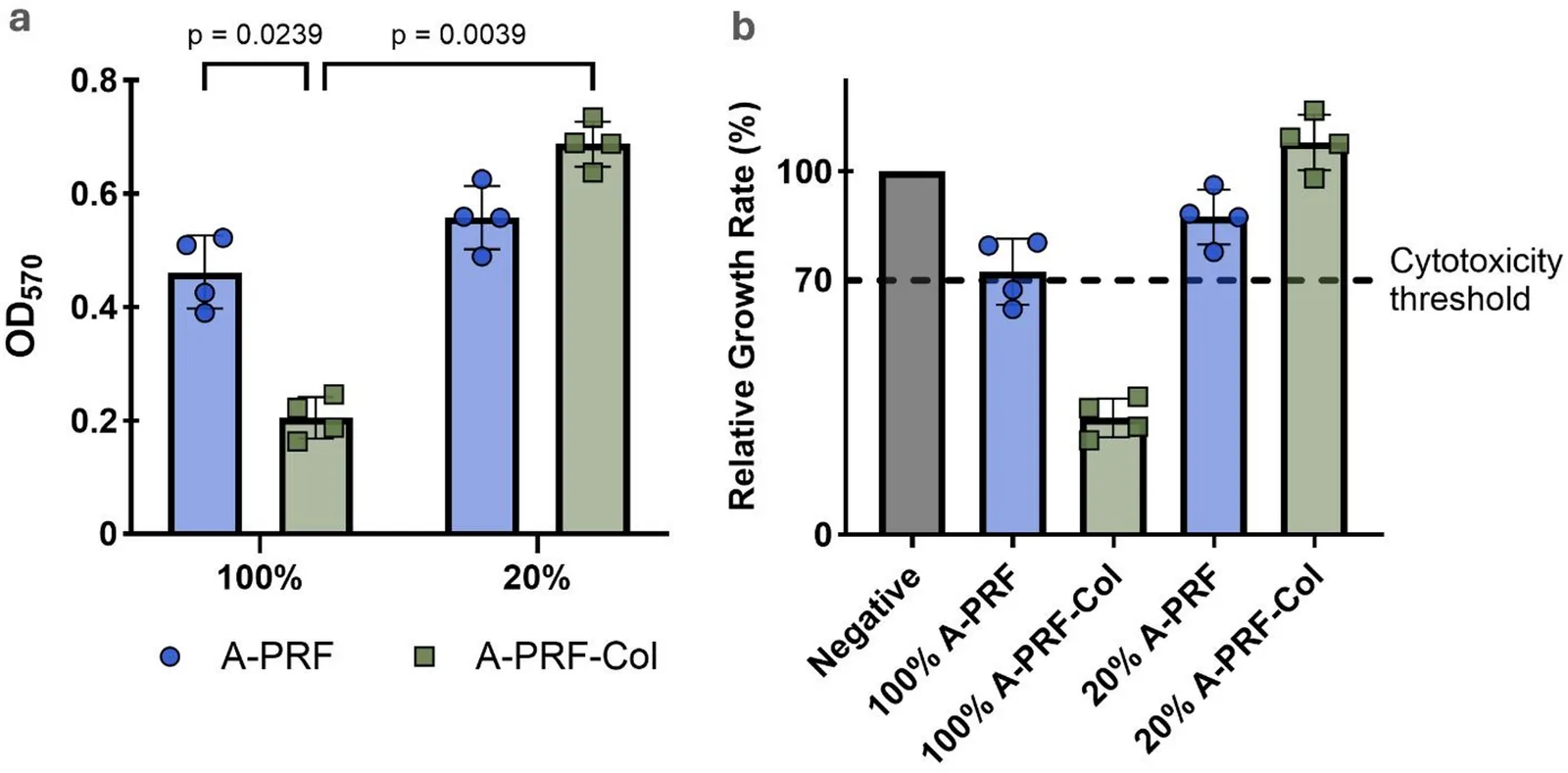
Effects of A-PRF and A-PRF-Col extracts on hPDLSC viability at 24 h. **a** Blank-corrected OD_570_ values of hPDLSCs after 24 h exposure to 100% and 20% A-PRF or A-PRF-Col extracts. Data were analysed using two-way repeated-measures ANOVA, followed by Šídák-adjusted pairwise comparisons. **b** Donor-level RGR percentages normalised to the negative control. The dashed line indicates the predefined 70% cytotoxicity threshold. Data are presented as mean ± standard deviation with individual donor-level values (*n* = 4)

After 24 h of exposure, analysis of blank-corrected OD_570_ values in the four experimental extract groups showed a significant main effect of concentration (*p* = 0.0009), no significant main effect of formulation (*p* = 0.0785), and a significant concentration × formulation interaction (*p* = 0.0101) (Fig. 1a). Šídák-adjusted pairwise comparisons showed that, at 100% concentration, A-PRF-Col resulted in significantly lower OD_570_ values than A-PRF (*p* = 0.0239). No significant difference between A-PRF and A-PRF-Col was observed at 20% concentration (*p* = 0.1361). Within the A-PRF condition, the difference between 100% and 20% extracts was not significant (*p* = 0.2498), whereas within the A-PRF-Col condition, OD_570_ values were significantly lower at 100% than at 20% concentration (*p* = 0.0039).

For descriptive cytotoxicity interpretation, the mean RGR values were 72.43 ± 9.08% for 100% A-PRF, 32.12 ± 5.30% for 100% A-PRF-Col, 87.53 ± 7.55% for 20% A-PRF, and 108.01 ± 7.69% for 20% A-PRF-Col (Fig. 1b). Based on mean RGR values, only 100% A-PRF-Col fell below the predefined 70% cytotoxicity threshold. However, individual donor-level RGR values for 100% A-PRF were 62.13%, 67.49%, 79.67%, and 80.45%, indicating that two of four donor-derived extracts were below the 70% threshold. Donor-level blank-corrected OD_570_ and RGR values are provided in Supplementary Table S1.

**Fig. 2:**
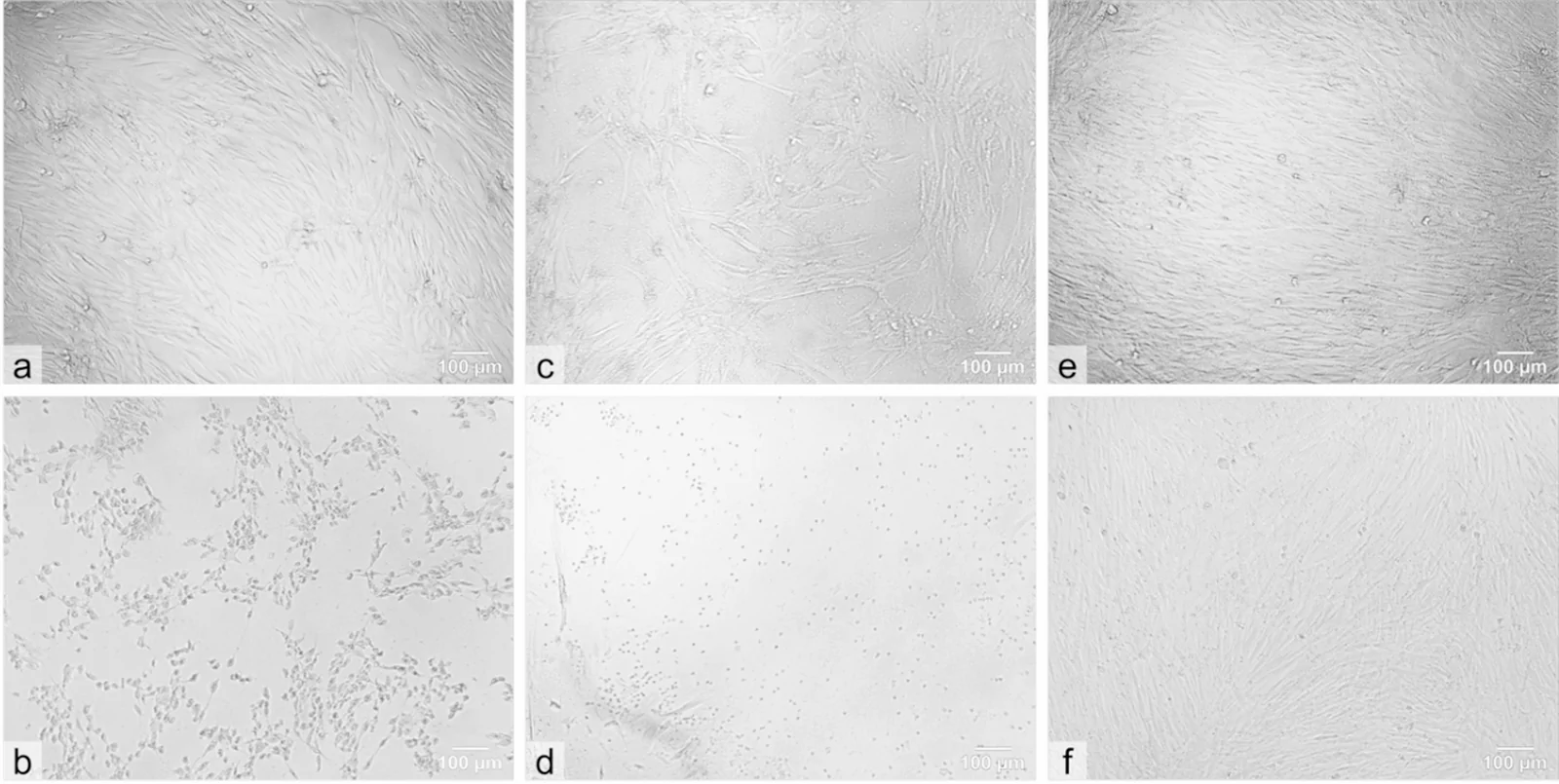
Microscopic morphologies of hPDLSCs after 24 h in the viability assay. Representative unstained microscopic images were captured before MTT incubation at 10× objective magnification. (**a**) Negative control; (**b**) positive control; (**c**) 100% A-PRF; (**d**) 100% A-PRF-Col; (**e**) 20% A-PRF; and (**f**) 20% A-PRF-Col. Scale bar = 100 μm

Qualitative microscopic observations were generally consistent with the MTT-based viability findings (Fig. 2). The negative control showed a dense adherent layer of elongated spindle-shaped cells (Fig. 2a), whereas the positive control showed marked deterioration, with many rounded, shrunken, clustered, or poorly attached cells and loss of typical spindle-shaped morphology (Fig. 2b). In the 100% A-PRF group, adherent elongated cells were still present, although the cell layer appeared less dense and less uniform than in the negative control (Fig. 2c). The 100% A-PRF-Col group showed the most pronounced adverse changes, with markedly reduced adherent cell density, sparse elongated cells, and numerous rounded cells or debris-like particles (Fig. 2d). In both 20% extract groups, most cells retained elongated adherent morphology broadly resembling the negative control (Fig. 2e, f). The 20% A-PRF group showed a dense spindle-shaped cell layer, while the 20% A-PRF-Col group also showed preserved attachment and elongated morphology.

### Effect of A-PRF and A-PRF-Col extracts on hPDLSC proliferation-related metabolic activity

**Fig. 3:**
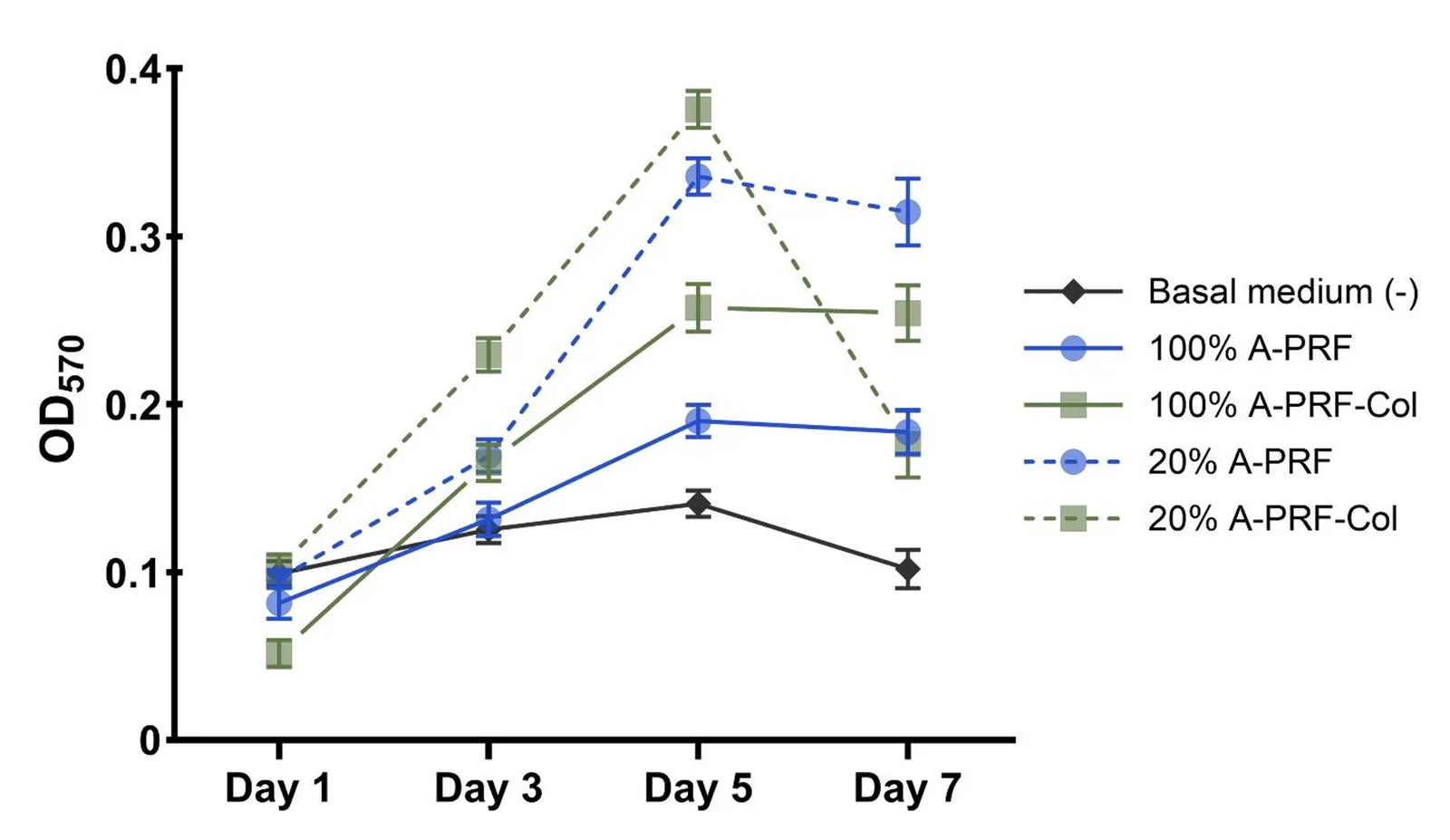
Effects of A-PRF and A-PRF-Col extracts on hPDLSC proliferation-related metabolic activity. Blank-corrected OD570 values were measured on days 1, 3, 5, and 7 after exposure to 100% and 20% A-PRF or A-PRF-Col extracts. Basal serum-free DMEM/F12 (-) is shown as a serum-free baseline reference control. Data are presented as mean ± standard deviation of donor-level values (*n* = 4)

MTT-derived proliferation-related metabolic activity changed over time in all four experimental groups, generally increased up to day 5, and showed group-specific patterns by day 7 (Fig. 3). Three-way repeated-measures ANOVA showed significant main effects of time (*p* < 0.0001) and concentration (*p* = 0.0017), but not formulation (*p* = 0.0852). All tested two-way and three-way interaction terms were significant. Detailed ANOVA results and prespecified pairwise comparisons are provided in Supplementary Table S2.

At 100% concentration, A-PRF-Col showed lower metabolic activity than A-PRF on day 1, but higher activity on days 3, 5, and 7. At 20% concentration, A-PRF-Col showed higher activity than A-PRF on days 3 and 5, no significant difference on day 1, and lower activity on day 7. Within the A-PRF formulation, the 20% extract showed higher metabolic activity than the 100% extract at all time points. Within the A-PRF-Col formulation, the 20% extract showed higher activity on days 1, 3, and 5, whereas the 100% extract showed higher activity on day 7.

In the secondary analysis, including the serum-free baseline reference control (-), significant effects of time, condition, and time × condition interaction were observed (all *p* < 0.0001; Supplementary Table S3). Dunnett-adjusted comparisons showed that, on day 1, 100% A-PRF and 100% A-PRF-Col had lower metabolic activity than the serum-free baseline reference control, whereas both 20% extracts were comparable with it. On day 3, 100% A-PRF remained comparable with the serum-free baseline reference control, while 100% A-PRF-Col and both 20% extracts showed higher metabolic activity. By days 5 and 7, all four experimental extract groups showed higher metabolic activity than the serum-free baseline reference control.

### Effect of A-PRF and A-PRF-Col extracts on hPDLSC migration-related wound closure

**Fig. 4:**
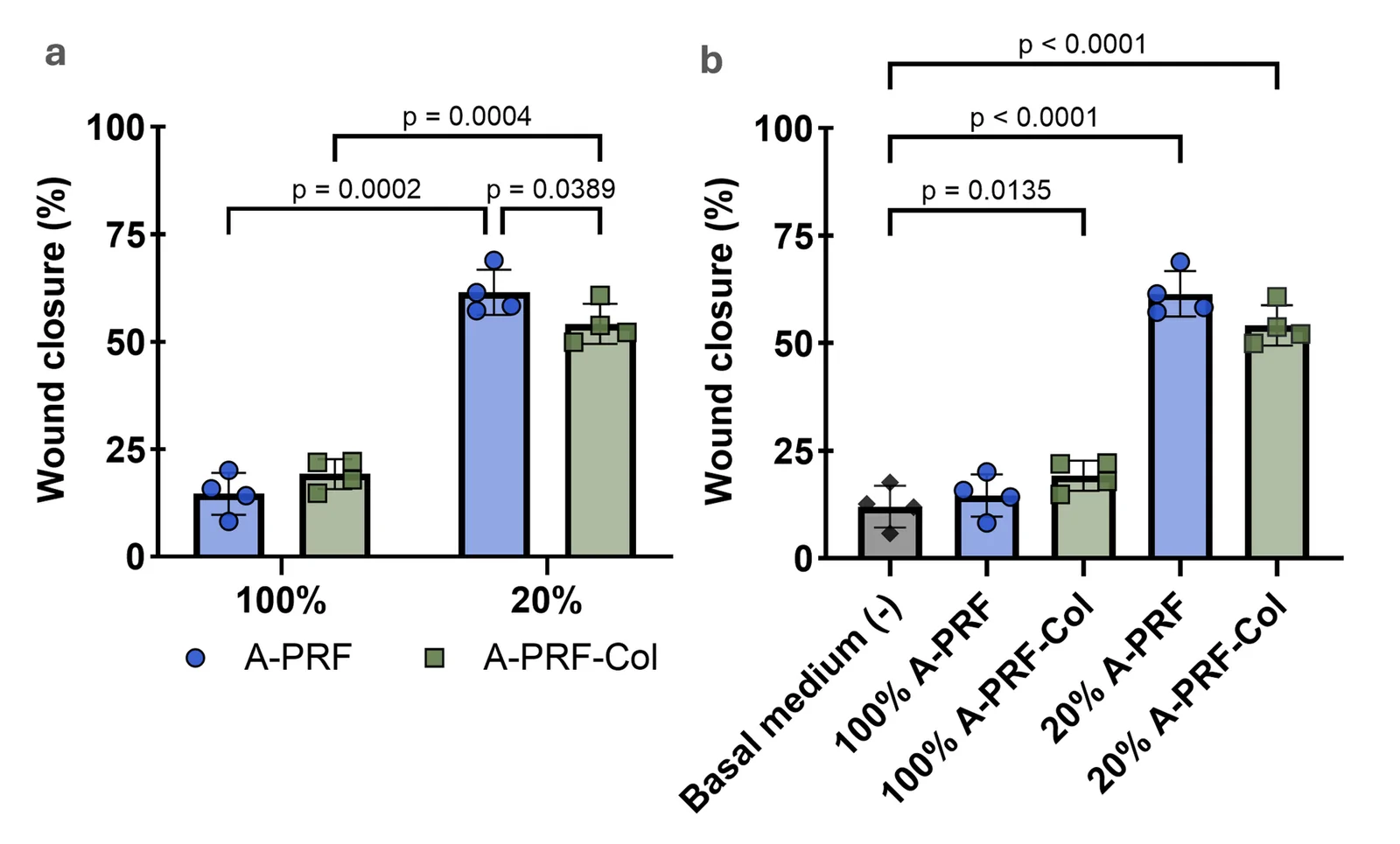
Effects of A-PRF and A-PRF-Col extracts on hPDLSC migration-related wound closure after 24 h. **a** Donor-level wound closure percentages in the four groups. Data were analysed using two-way repeated-measures ANOVA with formulation and concentration as within-donor factors, followed by Šídák-adjusted pairwise comparisons. **b** Donor-level wound closure percentages, including the serum-free baseline reference control, were analysed using repeated-measures one-way ANOVA followed by Dunnett’s multiple-comparison test versus the serum-free baseline reference control. Data are presented as mean ± standard deviation with individual donor-level values (*n* = 4)

Migration-related wound closure after 24 h was mainly associated with extract concentration (Fig. 4a). Two-way repeated-measures ANOVA of the four experimental groups showed a significant main effect of concentration (*p* = 0.0004), no significant main effect of formulation (*p* = 0.3369), and a significant concentration × formulation interaction (*p* = 0.0134). Detailed statistical outputs for the primary and secondary migration-related wound closure analyses are provided in Supplementary Table S4.

In both formulations, the 20% extracts produced greater wound closure than their corresponding 100% extracts. At 20% concentration, A-PRF induced greater wound closure than A-PRF-Col, whereas no significant difference between formulations was observed at 100% concentration.

In the secondary reference-control analysis, a significant overall group effect was observed (*p* < 0.0001) (Fig. 4b). Compared with the serum-free baseline reference control, 100% A-PRF did not significantly increase wound closure, whereas 100% A-PRF-Col produced a modest but significant increase. Both 20% A-PRF and 20% A-PRF-Col significantly increased wound closure relative to the serum-free baseline reference control.

**Fig. 5:**
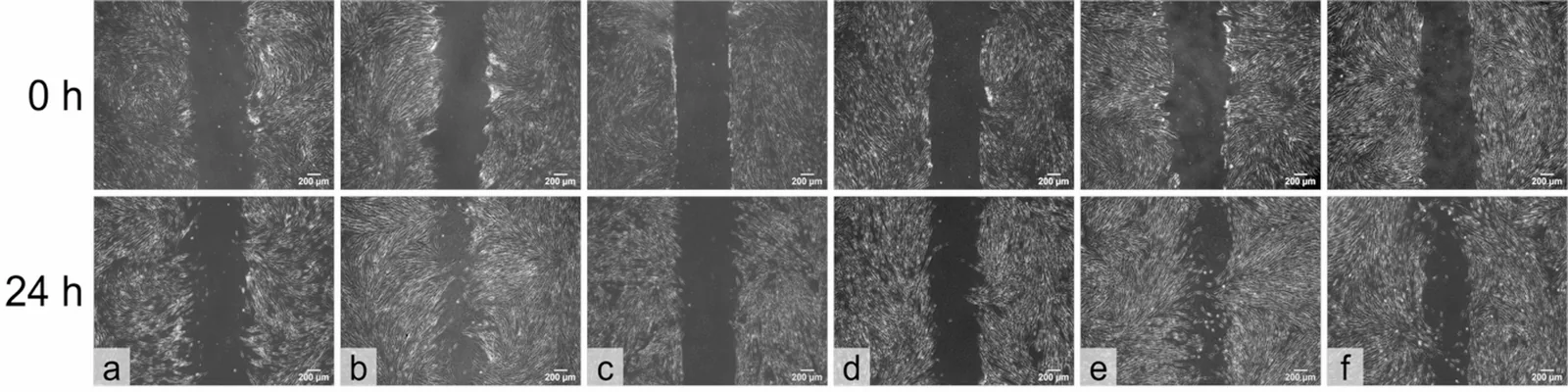
Representative scratch assay images of hPDLSCs at 0 and 24 h. Representative images from one donor-derived extract are shown. For each condition, the same pre-marked field was imaged at 0 h and 24 h. (**a**) Serum-free baseline reference control; (**b**) Growth-supporting reference control; (**c**) 100% A-PRF; (**d**) 100% A-PRF-Col; (**e**) 20% A-PRF; and (**f**) 20% A-PRF-Col. Scale bar = 200 μm

Representative scratch images were consistent with the quantitative findings (Fig. 5). After 24 h, the cell-free gap remained relatively wide in the serum-free baseline reference control and in both 100% extract groups. In contrast, the gap narrowed more clearly in the growth-supporting reference control and in both 20% extract groups. Among the experimental groups, 20% A-PRF showed the greatest apparent wound closure, followed by 20% A-PRF-Col, whereas the difference between 100% A-PRF and 100% A-PRF-Col appeared less pronounced.

## Discussion

In this exploratory in vitro extract model, early hPDLSC responses to A-PRF differed between the two tested extract concentrations and were further modified when A-PRF was combined with a collagen membrane. Overall, the 20% extracts elicited more favourable early cellular responses than the 100% extracts. The influence of the collagen membrane was endpoint-specific rather than uniformly beneficial. The 100% A-PRF-Col group showed the least favourable viability profile, 20% A-PRF-Col showed higher proliferation-related metabolic activity at intermediate time points, and 20% A-PRF showed the greatest migration-related wound closure. These findings suggest that A-PRF-Col should not be considered merely a stronger version of A-PRF, but rather a construct with an endpoint-specific and concentration-related biological profile.

The viability assay data indicate that adding a collagen membrane did not consistently improve the early cell compatibility of A-PRF, particularly at the undiluted extract concentration. Only 100% A-PRF-Col showed a mean RGR below the predefined 70% cytotoxicity threshold, consistent with the marked morphological deterioration observed microscopically. However, 100% A-PRF remained borderline and donor-variable, with two of four donor-derived extracts below 70%. Although PRF-based preparations often show favourable cellular effects in vitro, their responses vary with preparation protocol, concentration, target cell type, biomaterial context, and assay design [7, 8]. The reduced viability with 100% A-PRF-Col may reflect elevated levels of soluble mediators, altered release of A-PRF factors by collagen, soluble collagen components, or unmeasured factors such as pH and osmolality. These explanations remain hypothetical because these parameters were not measured.

For proliferation-related metabolic activity, the overall pattern favoured the 20% extracts, particularly during the first 5 days. All experimental groups increased until day 5, but their trends diverged afterwards. Within the A-PRF condition, 20% A-PRF consistently exceeded 100% A-PRF, whereas for the A-PRF-Col groups, 20% A-PRF-Col was higher at days 1, 3, and 5, but not at day 7. These data suggest that a lower extract concentration may provide a more favourable environment for early cellular metabolic activity. At the same time, the collagen membrane may affect the timing of this response rather than producing a uniform enhancement. In the secondary control-based analysis, both 100% extracts were lower than the serum-free baseline reference control on day 1. In contrast, all experimental groups showed higher metabolic activity than the serum-free baseline reference control by days 5 and 7. These findings should be interpreted in the context of an MTT-based assay conducted under sustained exposure to serum-free extracts. Thus, they specifically indicate proliferation-related metabolic activity, rather than direct cell proliferation or cell number.

These findings align with our previous study, in which exudates from A-PRF combined with a xenogenic bone substitute material promoted hPDLSC proliferation, with lower concentrations outperforming the 100% concentration at several time points and showing a general peak around day 5 [18]. In contrast, Li et al. reported that 100% and 20% PRF exudates promoted stronger hPDLC proliferation than 4%, suggesting a dose-related stimulatory pattern in their model [15]. This difference is plausible because the PRF protocol, biomaterial context, extract preparation, target cells, and assay design differed across studies [7, 15, 18]. Other studies also support the view that PRF-related cellular effects are protocol- and endpoint-dependent. Thanasrisuebwong et al. showed that red and yellow i-PRF exerted differential effects on PDLSC proliferation, migration, and osteogenic differentiation [16]. At the same time, Zheng et al. reported that liquid PRF promoted hPDLC proliferation, migration, osteogenic differentiation, and modulation of inflammation [17]. More recently, Dohle et al. found that PRF treatment of human primary periodontal ligament cells may suppress proliferation while promoting osteogenic differentiation-related outcomes [21]. Together, these studies indicate that PRF-based preparations do not necessarily produce uniformly stimulatory responses across all cellular endpoints.

The wound closure assays further support that extract concentration was the main factor associated with this outcome. Both A-PRF and A-PRF-Col induced greater migration-related wound closure at 20% than at 100%, and at 20% concentration, A-PRF showed greater closure than A-PRF-Col. These results suggest that the collagen membrane did not improve migration-related wound closure under the present conditions and may have attenuated the effect of A-PRF at the lower concentration. One possible explanation is that migration-relevant soluble mediators released from A-PRF may have been retained, adsorbed, or released differently after contact with the collagen membrane, reducing their availability in the final extract. This remains speculative because growth factor release, cytokine profiles, and mediator retention were not measured. In addition, scratch closure reflects a composite response influenced by collective migration, cell spreading, and proliferation-related activity. Although pre-marked fields and technical replicate wells were used to improve consistency, analysing one field per well may not fully capture within-well heterogeneity caused by manually created scratches. Therefore, the present outcome should be interpreted as migration-related wound closure rather than direct migration alone.

The differing responses across the three assays may reflect the fact that A-PRF-based extracts act through more than the amount of released bioactive mediators alone. Release timing, mediator retention, extract concentration, serum-free exposure, and physicochemical properties may all influence cell responses. A-PRF has been reported to support growth factor release and cellular responses within the low-speed centrifugation concept [6, 8]. Still, the present findings suggest that maximal extract concentration is not necessarily optimal. This may explain why the 20% extracts generally produced more favourable early responses than the 100% extracts.

The role of the collagen membrane likely involves changing how A-PRF interacts with the biomaterial and how A-PRF-derived signals are presented in the extract. Collagen membranes are not biologically inert carriers, and their physicochemical properties can influence liquid uptake, penetration, retention, and release kinetics [3, 5]. Al-Maawi et al. showed that liquid PRF interacted differently with collagen-based biomaterials: it completely invaded Mucograft^®^, partly invaded Bio-Gide^®^ and Mucoderm^®^, showed superficial interaction with Collprotect^®^, and showed minimal interaction with BEGO^®^ collagen membrane [9]. Thus, Bio-Gide^®^ should be interpreted as showing partial penetration rather than minimal absorption in that study. Similarly, Blatt et al. reported that PRF-biofunctionalised collagen matrices showed increased early growth factor release and angiogenesis-related responses [10], and Park et al. found that PRF modification of a porcine-derived collagen matrix improved endothelial cell proliferation, migration, and attachment in vitro [11]. These studies indicate that collagen-based biomaterials can affect how PRF-derived signals are presented or released. However, because this evidence largely comes from liquid PRF or PRF-biofunctionalised collagen matrices, it should be extrapolated cautiously to the present solid A-PRF-Col extract model. Because growth factor release, cytokine profiles, and mediator retention were not measured in this study, the proposed explanation remains hypothetical.

The difference between the viability assay and scratch assay in the 100% A-PRF-Col group should also be interpreted carefully. Although 100% A-PRF-Col showed the lowest RGR and the least favourable morphology after 24 h in the viability assay, it still produced a modest but significant increase in wound closure compared with the serum-free baseline reference control. This is not necessarily contradictory, as the assays evaluated different aspects of cell behaviour across different experimental settings. Limited wound closure may still occur through migration-related movement and cell spreading, even when overall viability-related metabolic activity is less favourable. This supports the broader interpretation that PRF-based systems may affect different regenerative-related endpoints in different directions [7, 16, 21].

From a translational perspective, the present findings suggest that PRF-based membrane constructs should not be assumed to perform better simply by increasing extract concentration or by adding a collagen membrane. Collagen membranes are widely used as barrier materials in guided tissue regeneration, but conventional membranes may have limited intrinsic bioactivity [3, 5]. Combining A-PRF with a collagen membrane is therefore biologically plausible. Still, the present data indicate that the construct requires further optimisation to balance early cell compatibility, proliferation-related metabolic activity, and migration-related wound closure.

Several limitations should be acknowledged. First, no collagen membrane-only extract group was included. Therefore, the effects of collagen alone could not be separated from those of the A-PRF-Col construct, and differences between A-PRF and A-PRF-Col cannot be attributed specifically to collagen-derived components, A-PRF-collagen interactions, or altered extract properties. Second, pH and osmolality were not measured, even though these factors may have contributed to the concentration-related differences observed between the 100% and 20% extracts. Third, growth factors, cytokines, release kinetics, signalling pathways, and gene-expression profiles were not analysed; therefore, the proposed explanations for reduced viability in 100% A-PRF-Col and attenuated wound closure in 20% A-PRF-Col remain hypothetical. Fourth, a single previously characterised hPDLSC donor-derived cell source was used. This reduced cell-source variability across experimental conditions but limits generalisability to hPDLSCs from other donors. Fifth, MTT assays reflect metabolic activity rather than direct cell number, and scratch closure reflects a composite response rather than direct migration alone. Finally, this in vitro extract model does not reproduce the vascular, immune, mechanical, and multicellular complexity of periodontal wound healing and cannot demonstrate periodontal regeneration or clinical efficacy.

Future studies should include collagen membrane-only extracts, a broader range of extract concentrations, and hPDLSCs from multiple donors. Direct measurement of pH and osmolality should be incorporated to determine whether physicochemical extract properties contribute to the observed concentration-related effects. Growth factor and cytokine profiling, release-kinetic studies, and gene-expression or signalling analyses would help clarify whether the collagen membrane modifies the availability of A-PRF-derived mediators. More complex three-dimensional, co-culture, and in vivo models will also be needed before the biological relevance of A-PRF-Col constructs for periodontal regeneration can be interpreted with confidence.

Taken together, these findings indicate that A-PRF-Col produced endpoint-specific responses rather than a uniform improvement over A-PRF. This supports further optimisation of A-PRF-collagen membrane preparation conditions before biological or clinical advantages are inferred.

## Conclusions

Within the present cell-source and extract-preparation conditions, this in vitro study showed that hPDLSC responses differed between A-PRF and A-PRF-Col and varied between the two tested extract concentrations. Diluted extracts generally elicited more favourable early cellular responses than undiluted extracts. The A-PRF-Col construct did not uniformly enhance A-PRF effects across endpoints but did exhibit an endpoint-specific, concentration-related biological profile. These exploratory findings support further optimisation of A-PRF-collagen membrane constructs before their regenerative or clinical advantages are inferred.

## Supplementary Information

Supplementary Material 1. Supplementary Table S1. Donor-level hPDLSC viability data after 24 h exposure to A-PRF and A-PRF-Col extracts. Supplementary Table S2. Primary statistical analysis of hPDLSC proliferation-related metabolic activity. Supplementary Table S3. Secondary control-based analysis of hPDLSC proliferation-related metabolic activity. Supplementary Table S4. Statistical summary of hPDLSC migration-related wound closure after 24 h.

## Data Availability

The data supporting the findings of this study are included in this article and its additional file. Additional datasets are available from the corresponding author on reasonable request.

## Abbreviations

A-PRF: Advanced platelet-rich fibrin
A-PRF-Col: Advanced platelet-rich fibrin combined with a collagen membrane
DMEM/F12: Dulbecco’s Modified Eagle’s Medium/Nutrient Mixture F-12
hPDLSCs: Human periodontal ligament stem cells
MTT: 3-(4,5-dimethylthiazol-2-yl)-2,5-diphenyltetrazolium bromide
OD: Optical density
RGR: Relative growth rate
