# Supplementary material for: Effects of advanced platelet-rich fibrin combined with a collagen membrane on human periodontal ligament stem cells at two extract concentrations: an in vitro study
Source: BMC Oral Health. 2026 Jun 26;26:1707. doi: 10.1186/s12903-026-09064-1 (PMC13563724; doi:10.1186/s12903-026-09064-1)
Supplement: Supplementary file 1 [file 12903_2026_9064_MOESM1_ESM.pdf]

Supplementary Table S1. Donor-level hPDLSC viability data after 24 h exposure to A-PRF and A-PRF-Col extracts.

A. Blank-corrected OD_{570} values

Group	Donor 1	Donor 2	Donor 3	Donor 4	Mean ± SD
100% A-PRF	0.557	0.544	0.460	0.425	0.497 ± 0.064
100% A-PRF-Col	0.222	0.257	0.198	0.281	0.240 ± 0.037
20% A-PRF	0.524	0.592	0.660	0.594	0.593 ± 0.056
20% A-PRF-Col	0.723	0.769	0.672	0.724	0.722 ± 0.040

B. Relative growth rate values (%)

Group	Donor 1	Donor 2	Donor 3	Donor 4	Mean ± SD
100% A-PRF	62.13	67.49	79.67	80.45	72.43 ± 9.08
100% A-PRF-Col	26.01	29.74	34.79	37.95	32.12 ± 5.29
20% A-PRF	77.88	88.42	87.49	96.30	87.53 ± 7.55
20% A-PRF-Col	116.87	109.36	107.66	98.15	108.01 ± 7.69

Values represent donor-level means, averaged across four technical replicates. RGR was calculated from blank-corrected OD_{570} values and normalised to the negative control. RGR values were used to interpret cytotoxicity descriptively, according to the predefined 70% threshold. Inferential analysis for viability was performed using blank-corrected OD_{570} values.

Supplementary Table S2. Primary statistical analysis of hPDLSC proliferation-related metabolic activity.

A. Three-way repeated-measures ANOVA

**Effect**	**F (DFn, DFd)**	**p value**
Time	F (1.679, 5.037) = 1729	<0.0001
Concentration	F (0.520, 1.561) = 2314	0.0017
Formulation	F (0.332, 0.996) = 30.09	0.0852
Time × concentration	F (3.000, 9.000) = 244.4	<0.0001
Time × formulation	F (3.000, 9.000) = 137.6	<0.0001
Concentration × formulation	F (0.700, 2.099) = 36.76	0.0232
Time × concentration × formulation	F (0.464, 1.392) = 121.0	0.0222

B. Prespecified Šídák-adjusted pairwise comparisons

**Analysis set**	**Comparison**	**Day 1**	**Day 3**	**Day 5**	**Day 7**
Within 100% concentration	A-PRF-Col vs A-PRF	↓ 0.0024	↑ 0.0300	↑ 0.0019	↑ 0.0016
Within 20% concentration	A-PRF-Col vs A-PRF	NS 0.0951	↑ 0.0011	↑ 0.0090	↓ 0.0166
Within A-PRF formulation	20% vs 100%	↑ 0.0428	↑ 0.0331	↑ 0.0001	↑ 0.0047
Within A-PRF-Col formulation	20% vs 100%	↑ <0.0001	↑ 0.0083	↑ 0.0005	↓ 0.0185

Blank-corrected OD_{570} values were used for analysis. Technical replicate wells were averaged before analysis to obtain donor-level values. The model included four extract groups. GraphPad Prism did not assume sphericity, so Greenhouse–Geisser-corrected degrees of freedom and p-values are reported. Pairwise-comparison cells show Šídák-adjusted p values. Arrows indicate the direction of the first term in each comparison: ↑, first term higher; ↓, first term lower. NS, not statistically significant.

Supplementary Table S3. Secondary control-based analysis of hPDLSC proliferation-related metabolic activity.

A. Two-way repeated-measures ANOVA with the serum-free baseline reference control

**Effect**	**F (corrected DFn, DFd)**	**p value**
Time	F (1.768, 5.305) = 1509	<0.0001
Condition	F (1.370, 4.109) = 401.2	<0.0001
Time × condition	F (1.765, 5.294) = 214.6	<0.0001

B. Dunnett-adjusted comparisons versus serum-free baseline reference control

**Condition**	**Day 1**	**Day 3**	**Day 5**	**Day 7**
100% A-PRF	↓ 0.0018	NS 0.6114	↑ 0.0007	↑ 0.0010
100% A-PRF-Col	↓ <0.0001	↑ 0.0041	↑ 0.0001	↑ 0.0006
20% A-PRF	NS 0.2965	↑ 0.0002	↑ <0.0001	↑ 0.0014
20% A-PRF-Col	NS 0.1750	↑ 0.0001	↑ <0.0001	↑ 0.0015

The secondary model included the four groups and the serum-free baseline reference control. Blank-corrected OD_{570} values were analysed using two-way repeated-measures ANOVA with time and condition as within-donor factors. Dunnett-adjusted comparisons were performed at each time point, comparing each extract group with the serum-free baseline reference control. Arrows indicate the direction of the extract group relative to the serum-free baseline reference control: ↑, extract group higher; ↓, extract group lower. NS, not statistically significant.

Supplementary Table S4. Statistical summary of hPDLSC migration-related wound closure after 24 h.

A. Primary two-way repeated-measures ANOVA

**Effect**	**p value**
Concentration	0.0004
Formulation	0.3369
Concentration × formulation	0.0134

B. Prespecified Šídák-adjusted pairwise comparisons

**Analysis set**	**Comparison**	**Direction**	**Adjusted p value**
Within 100% concentration	A-PRF-Col vs A-PRF	NS	0.1238
Within 20% concentration	A-PRF-Col vs A-PRF	A-PRF higher	0.0389
Within A-PRF formulation	20% vs 100%	20% higher	0.0002
Within A-PRF-Col formulation	20% vs 100%	20% higher	0.0004

C. Secondary comparisons versus serum-free baseline reference control

**Condition**	**Direction versus control**	**Adjusted p value**
100% A-PRF	NS	0.5370
100% A-PRF-Col	Higher	0.0135
20% A-PRF	Higher	<0.0001
20% A-PRF-Col	Higher	<0.0001

Analyses were performed using donor-level wound closure percentages, averaged across four technical replicates. The primary model included only the four experimental extract groups. Pairwise comparisons were adjusted using the Šídák method. Secondary comparisons with the serum-free baseline reference control were adjusted using Dunnett’s multiple-comparison test. NS, not statistically significant.
